# Normobaric hyperoxia and the microcirculation in critically ill patients: a prospective observational study

**DOI:** 10.1186/cc14222

**Published:** 2015-03-16

**Authors:** S Zuccari, A Donati, E Damiani, E Montesi, S Ciucani, M Rogani, P Pelaia

**Affiliations:** 1 Università Politecnica delle Marche, Ancona, Italy

## Introduction

It is well known that oxygen acts as a vasoconstrictor. We evaluated the impact of normobaric hyperoxia on the sublingual microcirculation in critically ill patients.

## Methods

Forty mechanically ventilated (FiO_2_ ≤50%) patients with hemodynamic stability were enrolled in a prospective observational study. The first 20 patients underwent a 2-hour period of hyperoxia (FiO_2 _= 100%), and 20 patients were studied as controls (no FiO_2 _variations). The sublingual microcirculation (three sites) was evaluated with sidestream dark-field imaging at baseline (t0), after 2 hours of hyperoxia (t1), and 2 hours after return to baseline (t2). Continuous video recording was also performed during FiO_2_ variations on one and the same area (2-minute video).

## Results

**Figure 1:**
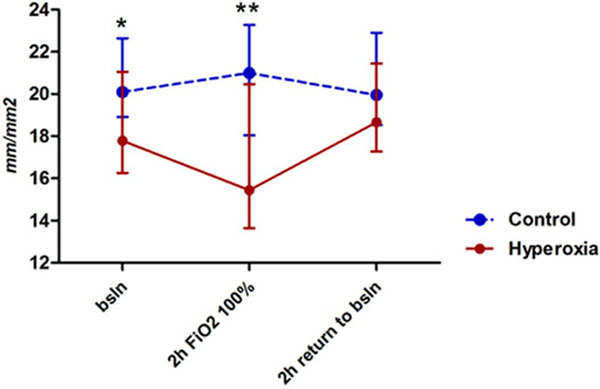


No changes in mean arterial pressure were observed. The perfused small vessel density tended to decrease at t1 and normalize at t2 (Figure 1) in the hyperoxia group. These variations appeared early after 2 minutes of FiO_2_ changes. A significant increase in lactate levels over time (from 1.1 (0.9 to 1.7) at t0 to 1.4 (1.1 to 1.9) mmol/l at t2, *P *= 0.01) was seen in the hyperoxia group.

## Conclusion

Hyperoxia induces an early decrease in microvascular perfusion, which appears to go back to normality at return to normoxia.

